# A pilot randomised controlled study of the mental health first aid eLearning course with UK medical students

**DOI:** 10.1186/s12909-018-1154-x

**Published:** 2018-03-21

**Authors:** E. Bethan Davies, Emmeline Beever, Cris Glazebrook

**Affiliations:** 1 Division of Psychiatry and Applied Psychology, School of Medicine, Institute of Mental Health, The University of Nottingham, Triumph Road, Nottingham, NG7 2TU UK; 2 NIHR MindTech MedTech Co-operative, Institute of Mental Health, The University of Nottingham, Triumph Road, Nottingham, NG7 2TU UK; 3 School of Medicine, The University of Nottingham, Queen’s Medical Centre, Nottingham, NG7 2UH UK

**Keywords:** Mental health first aid, Mental health literacy, Mental health, eLearning, Undergraduate, Stigma, Medical students, Health promotion

## Abstract

**Background:**

Medical students face many barriers to seeking out professional help for their mental health, including stigma relating to mental illness, and often prefer to seek support and advice from fellow students. Improving medical students’ mental health literacy and abilities to support someone experiencing a mental health problem could reduce barriers to help seeking and improve mental health in this population. Mental Health First Aid (MHFA) is an evidence-based intervention designed to improve mental health literacy and ability to respond to someone with a mental health problem. This pilot randomised controlled trial aims to evaluate the MHFA eLearning course in UK medical students.

**Methods:**

Fifty-five medical students were randomised to receive six weeks access to the MHFA eLearning course (*n* = 27) or to a no-access control group (*n* = 28). Both groups completed baseline (pre-randomisation) and follow-up (six weeks post-randomisation) online questionnaires measuring recognition of a mental health problem, mental health first aid intentions, confidence to help a friend experiencing a mental health problem, and stigmatising attitudes. Course feedback was gathered at follow-up.

**Results:**

More participants were lost follow-up in the MHFA group (51.9%) compared to control (21.4%). Both intention-to-treat (ITT) and non-ITT analyses showed that the MHFA intervention improved mental health first aid intentions (*p* = <.001) and decreased stigmatising attitudes towards people with mental health problems (*p* = .04). While ITT analysis found no significant Group x Time interaction for confidence to help a friend, the non-ITT analysis did show the intervention improved confidence to help a friend with mental health problems (*p* = <.001), and improved mental health knowledge (*p* = .003). Medical students in the intervention group reported a greater number of actual mental health first aid actions at follow-up (*p* = .006). Feedback about the MHFA course was generally positive, with participants stating it helped improve their knowledge and confidence to help someone.

**Conclusion:**

This pilot study demonstrated the potential for the MHFA eLearning course to improve UK medical students’ mental health first aid skills, confidence to help a friend and stigmatising attitudes. It could be useful in supporting their own and others’ mental health while studying and in their future healthcare careers.

**Trial registration:**

Retrospectively registered (ISRCTN11219848).

## Background

The mental health of medical students has received particular attention due to the stress associated with medical education and their roles as future healthcare professionals [1], and a recent meta-analysis estimates a mean depression prevalence rate of 28% in this group [2]. Studying medicine is highly demanding and is associated with a range of stressors, including academic and clinical assessments; changes in lifestyle and routine; financial pressures; and coping with emotionally-taxing situations [2, 3]. Untreated mental health problems can impact upon the skills students need to manage their workload, adversely affect their course attendance and engagement, and can lead to increased suicidal ideation and other harmful outcomes [2, 4].

Under-treatment of mental health problems is typical within student populations, and students often prefer to manage their own issues and to seek help from informal sources (e.g. family, friends) [5, 6]. Medical students face additional obstacles in seeking out help, both during their training and in their future working roles [7]: this includes perceived implications upon their career, fear of documentation, concerns about confidentiality, and greater time pressures [2, 7]. Stigma about mental health problems and help-seeking are important factors influencing medical students’ management of their mental health [4]. A qualitative study involving British medical students reported that the stigma associated with mental distress, feeling ashamed and embarrassment in having a “weakness” and how this would be perceived by academic staff, hindered help-seeking [8]. This has obvious potential implications for their management of mental health problems in themselves and for their patients. Medical students may have better mental health literacy and be able to provide better-quality support for peers experiencing depression given their degree, psychiatric training, and personal interests in health [2, 9, 10]. However recent findings report that one of the biggest challenges students face in supporting friends is knowing what to do and how they can help [11].

Mental Health First Aid (MHFA) is a structured course designed to improve trainees’ mental health literacy and abilities to assist themselves and someone developing a mental health problem or crisis [12]. Central to MHFA is an action plan (‘ALGEE’) applied to someone in need: **a**pproach the person, **a**ssess and **a**ssist with any crisis; **l**isten non-judgementally; **g**ive support and information; **e**ncourage the person to seek appropriate professional help; and **e**ncourage other support strategies (e.g. self-help) [12]. MHFA action plans have been developed through Delphi methodology for individual mental disorders (e.g. depression, psychosis) and mental health crisis situations (e.g. panic attacks, suicidal thoughts and behaviours) [13–16].

The Standard MHFA course is delivered in groups by an instructor over twelve hours, and has been tailored to different cultural, occupational, and age groups [17, 18]. A meta-analysis of 15 studies supports the effectiveness of MHFA in improving mental health literacy (Δ = 0.56), decreasing stigmatizing attitudes (Δ = 0.28) and improving actual MHFA behaviour (Δ = 0.25) [19]. The MHFA course has been recently adapted for eLearning delivery, including a tailored course for medical students [4]. eLearning is potentially more flexible and accessible to students [20], and online MHFA may also be more cost-effective as it does not require instructors or necessitate attending a specific location [21]. MHFA via e-Learning is as effective as face-to-face MHFA training in improving mental health first aid intentions, self-reported confidence in helping a friend, knowledge about mental health, and personal stigma towards mental illness [4, 21].

Previous evaluations of MHFA eLearning [4, 21, 22] and course evaluations with university students [4, 18, 23] have taken place in Australia. The present study aimed to pilot the MHFA eLearning course with UK medical students. We expected that students who received the MHFA eLearning course would report greater improvements in mental health first aid intentions, fewer stigmatising attitudes and more confidence in supporting a peer, compared to a control group.

## Methods

### Design

The study was a two-arm randomised controlled pilot study, with data collected at baseline (pre-randomisation) and six weeks follow-up, between October–December 2015. Participants meeting the inclusion criteria and consenting to participate were randomly allocated to intervention or control group via an online randomisation service [24]. Stratified allocation (in blocks of four) was used to ensure equivalent numbers of male and female participants in each group. This allocation was performed by EBD. The researcher responsible for outcome assessment and data analysis (EB) was blinded to allocation, but it was not possible to blind participants to group.

### Intervention: The MHFA eLearning course

Participants randomised to the intervention group received an email link to the MHFA eLearning course and informed that they would complete a follow-up questionnaire in 6 weeks’ time, followed by standardised reminder emails at two and four weeks. The MHFA eLearning course comprises six modules completed consecutively (Table 1). Content is delivered through text, images, audio, videos and interactive activities, and completed at the user’s own pace over approximately 6–8 h. Within each module, the users are educated about specific mental disorders and associated crises, and are taught the disorder-specific MHFA action plan. Participants also received a hard copy (via post) of the MHFA Manual, 3rd Edition [25] and a supplementary manual for medical students [26]. Some adjustments were made (by EBD) to the materials (e.g. colloquialisms, available mental health services) to make it more appropriate for British medical students. Participants completed the course at their own time and pace between October–December 2015.

**Table 1:** The content of the six modules within the MHFA eLearning course

Name of module	Content within module
1. Introduction to mental health and Mental Health First Aid	• Definitions, prevalence, and impact of mental health problems • Spectrum of interventions available for mental health problems • Types of professional help and treatment available for mental health problems • Definitions of recovery for mental health problems • Explanation of Mental Health First Aid and introduction to the MHFA ‘ALGEE’ action plan
2. Depression	• Definition of depression and other mood disorders • Risk factors for developing depression/mood disorders • Interventions and treatment for depression/mood disorders • Importance of early intervention for depression/mood disorders • Crises associated with depression: suicidal thoughts and behaviours, and non-suicidal self-injury • MHFA action plan for depression • Helpful resources list
3. Anxiety problems	• Definition of anxiety and anxiety disorders (e.g. PTSD, social anxiety disorder) • Risk factors for developing anxiety disorders • Interventions and treatment for anxiety disorders • Importance of early intervention for anxiety problems • Crises associated with anxiety problems: panic attacks, and traumatic events • MHFA action plan for anxiety • Helpful resources list
4. Eating disorders	• Definitions and types of eating disorders (e.g. anorexia nervosa, bulimia nervosa) • Risk factors for developing eating disorders • Interventions and treatment for eating disorders • Importance of early intervention for eating disorders • Crises associated with eating disorders: malnutrition-related crises, suicidal thoughts and behaviours, and non-suicidal self-injury • MHFA action plan for eating disorders • Helpful resources list
5. Psychosis	• Definition of psychosis and types of psychotic disorders (e.g. bipolar disorder, schizophrenia) • Risk factors for developing psychotic disorders • Interventions and treatment for psychotic disorders • Importance of early intervention for psychotic disorders • Crises associated with psychosis: person may be in severe psychotic state, aggressive behaviours, and suicidal thoughts and behaviours • MHFA action plan for psychosis • Helpful resources list
6. Substance use problems	• Definitions of substance use problems (e.g. alcohol use problems, drug use problems) • Risk factors for developing substance use problems • Interventions and treatment for substance use problems • Importance of early intervention for substance use problems • Crises associated with substance use problems: suicidal thoughts and behaviours, severe effects from alcohol and/or drug misuse (e.g. intoxication, withdrawal, overdosing, medical emergencies), and aggressive behaviours • MHFA action plan for substance use problems • Helpful resources list

### Control

Participants randomised to control group received an email informing them that they would be contacted in six weeks’ time to complete the follow-up questionnaire.

### Ethical approval

The study was reviewed and approved by the University of Nottingham (UoN) Medical and Health Sciences research ethics committee (ref: T14072015). Informed consent was obtained from participants through ticking several boxes in the baseline online questionnaire.

### Participants and recruitment

Medical students (aged ≥18 years) studying undergraduate or graduate entry medicine at UoN and who were in their first, second or third year of study, were eligible to participate. As is typical in the UK, most participants in this study had commenced their five-year medical course at the age of 18 years, directly after completing further education. The University of Nottingham also offers a four-year, graduate-entry medical course, which admits older students who already have a university degree and, often, previous vocational experience. Students in the early years of the two medical courses have had some education around mental illness but would not yet have had their psychiatry attachment. Participants needed to have regular access to the internet and to a laptop/computer, and were recruited during October 2015 through a range of media including the university’s web-based learning platform and Twitter feed, e-newsletters, undergraduate lectures, and posters placed around the medical school. These advertised the study as an opportunity to undertake an online MHFA course which would take approximately eight hours to complete. Advertisements explained that the course was designed to help people provide support to someone experiencing a mental health problem or crisis, and all advertisements signposted students towards an online eligibility questionnaire. Advertisements also included an incentive (to enter into a prize draw to win one of ten retail vouchers) upon finishing the study (i.e. completion of the follow-up survey). Participants’ overall study involvement was 6–8 weeks.

### Power calculation

The minimum target sample size for this pilot study was 50 students. Based on previous research which found a mean MHFA intentions score of 3.45 (±1.67) in a similar population [10] it was estimated that a sample size of 50 (25 in each group) would have 80% power to detect a 1.33 point difference between the groups.

At baseline, participants completed questions about their age, gender, year of study, whether they were currently on placement. They completed a battery of outcome measures (described below) at baseline and follow-up: these were selected from previous evaluations of MHFA [19].

### Procedure and outcome measures

Interested participants completed the online eligibility questionnaire. Eligible participants were provided with detailed information about the study and signposted towards the online baseline questionnaire. After completing the baseline measures, participants were randomised to intervention or control, and informed that they would receive an online follow-up questionnaire in six weeks’ time. After completing the follow-up questionnaire, participants were presented with a debriefing webpage and could enter an optional prize draw. All participants were emailed detailed information about university services and local, national and online mental health resources.

### Experience of mental health problems and mental health-related course curricula

Participants’ current level of psychological distress was self-reported through the Depression Anxiety and Stress Scale, 21-item version (DASS-21) [27]. This consists of 21 items (divided into three subscales) measuring occurrence of depression, anxiety and stress symptoms within the prior week, with each item scored on a four-point Likert scale ranging from 0 (“did not apply to me at all”) to 3 (“applied to me very much, or most of the time”). Total scores are multiplied by two to allow for comparison to the original 42-item DASS scale. Previous administration of DASS-21 in student populations has found high internal consistency for the three subscales [28, 29]. Higher scores, both overall and on each subscale, indicate poorer mental health.

Five questions explored experience of mental health problems: 1) self-reported personal experience of a mental health problem, and if so whether they had sought help; 2) whether they had a close friend or relative experience a mental health problem; 3) self-reported exposure to media campaigns in the past 12 months; 4) whether they had taken any psychiatry-related modules or placements during their degree; 5) any previous experience of the MHFA course. All questions were presented with three answer options (“Yes”, “No”, “Unsure”).

### Mental health knowledge

Before and after completing the course, participants randomised to intervention completed 20 true or false questions based on content within the MHFA eLearning course [4], e.g. “If someone has a traumatic experience, it is best to make them talk about it as soon as possible”. This quiz was embedded within the MHFA eLearning platform.

### Recognition of a mental health problem and mental health first aid intentions

Participants were quasi-randomised (by month of birth) to read a text vignette describing a 21 year-old male university student (‘Mark’) experiencing symptoms of DSM-IV criteria for either depression or social anxiety/phobia [30]. After viewing the vignette, they were asked to identify the main problem depicted in the vignette with an open-ended question: *‘What, if anything, would you say is Mark’s main problem?’* [31]. Answers were coded as ‘correct’ if they mentioned “depression” if they saw the depression vignette, or “social anxiety” or “social phobia” if they saw the social anxiety/phobia vignette [4, 18].

To assess mental health first aid intentions towards the vignette, participants answered an open-ended question: ‘Imagine Mark is someone you have known for a long time and care about. You want to help him. What would you do?’ [30]. Participants’ qualitative responses were coded using a scoring scheme based on the MHFA action plan [32]. Responses are coded for each ‘ALGEE’ component mentioned in the response (i.e. approach the person; assess and assist with any crisis; listen non-judgementally; give support and information; encourage appropriate professional help; and encourage other support). A score of ‘0′ indicates the component was not mentioned; ‘1′ is a helpful but superficial response; and ‘2′ is a good response with relevant specific detail. Scores from each component were summed to produce a total intentions score ranging from 0 to 12. Higher scores indicated better quality mental health first aid intentions [33].

All qualitative responses were coded by one rater (EB, blind to intervention condition) with discussion with a second trained rater (EBD). To establish coding reliability, EB coded 60 responses from a previous study [34] and compared to consensus codes (coded by three experts in MHFA) using intra-class correlations (ICC). The degree of matching was moderate-to-high (approach the person = .386; assist and assess crisis = .901; listen non-judgementally = .714; give support and information = .703; encourage professional help = .857; encourage other supports = .466; all significant at *p = .001 level).*

### Confidence to help a friend experiencing a mental health problem

Participants self-rated their confidence in their ability to help a friend experiencing symptoms similar to the vignette on a four-point Likert scale, ranging from “not confident at all” to “very confident”, or could select a “don’t know” option [35].

### Actual mental health first aid actions taken

Participants self-reported how many times (“never”, “once”, “a few times”, many times they had spoken with a close other (e.g. friend, relative) about their mental health problem in the past six months (at baseline) and past six weeks (at follow-up) [22]. Participants were asked to indicate what actions they had taken through selecting from a list of nine actions presented on a checklist, which included a free-text box to enter additional actions [22].

### Stigma towards mental illness

The personal stigma subscale of the Depression Stigma Scale (DSS), adapted for young people, was used to assess the participants’ stigmatising attitudes towards the student depected in the vignette (e.g. “Mark’s problem is not a real medical illness”) [36, 37]. The personal stigma subscale consists of seven items, scored on a five-point Likert scale from ‘strongly disagree’ to ‘strongly agree’, with a range from 0 to 28. Higher scores indicate greater personal stigma. Previous research in a similar population reported excellent internal consistency (α = 0.81) for this subscale [10], with the present study calculating α = .86.

### Feedback about participation and the MHFA eLearning course

At follow-up all participants were asked their motivations for participating in the study and presented with a multiple choice checklist of seven possible reasons for participation, alongside a free-text box for additional comments. Intervention group participants also completed a series of rating scales and open-ended questions to gather their opinions about the MHFA eLearning course. This was guided by previous evaluation of MHFA [4].

### Data analysis

Analyses were carried out using SPSS V.22 (Chicago, IL). Baseline differences between the two groups were explored through t-tests, Mann Whitney U tests, and Chi Square tests. Repeated measures ANOVAs were performed with the three scale-based measures (mental health first aid intentions, confidence to help a friend, personal stigma), with the measure inputted as the dependent variable, group (intervention/control) as the between-subjects factor, and time (baseline/follow-up) as the within-subjects factor. McNemar’s Tests explored pre-post differences in participants’ recognition of a mental health problem. Intention-to-treat (ITT) and non-ITT analyses were performed for each outcome measure. For ITT, participants who did not complete the follow-up questionnaire had their last observation (i.e. baseline measure) carried forward. Non-ITT analyses were only performed on participants who completed baseline and follow-up questionnaires (i.e. responders). ITT analysis was not performed for the actual mental health first aid actions outcome measure, as the baseline item used a different time anchor to follow-up (i.e. past six months at baseline, past six weeks at follow-up). Content analysis was used to analyse qualitative feedback about the MHFA eLearning course.

## Results

### Demographics

Of 144 medical students who completed the eligibility questionnaire, 131 were eligible to participate in the study. Of these, 55 students consented to participate and completed the baseline questionnaire and were randomised to intervention or control. The intervention and control group were well matched at baseline in terms of demographic characteristics, current mental health, and mental health first aid outcomes, with no significant differences between the groups (all *p* = > .05). Table 2 shows the characteristics of both groups at baseline.

**Table 2:** Characteristics of the intervention and control groups at baseline

	MHFA eLearning (*n* = 27) N (%)	Control (*n* = 28) N (%)
Gender		
Male	9 (33.3)	10 (35.7)
Female	18 (66.7)	18 (64.3)
Age (M, SD)	20.3 (4.42)	19.4 (1.25)
Year of study		
1st Year	10 (37.0)	7 (25)
2nd Year	15 (55.6)	15 (53.6)
3rd Year	2 (7.4)	6 (21.4)
Depression symptoms (M, SD)	8.81 (9.46)	6.21 (6.21)
Anxiety symptoms (M, SD)	5.70 (6.09)	5.78 (7.39)
Stress symptoms (M, SD)	10.88 (8.17)	8.00 (7.73)
Personal experience of mental health problem	8 (29.6)	6 (17.9)
Close friend/relative experienced a mental health problem	19 (70.4)	21 (75)
Exposure to mental health campaigns in past 12 months	23 (85.2)	24 (85.7)
Talked to someone about their mental health in the past 6 months	19 (70.3%)	18 (64.3%)

**Fig. 1:**
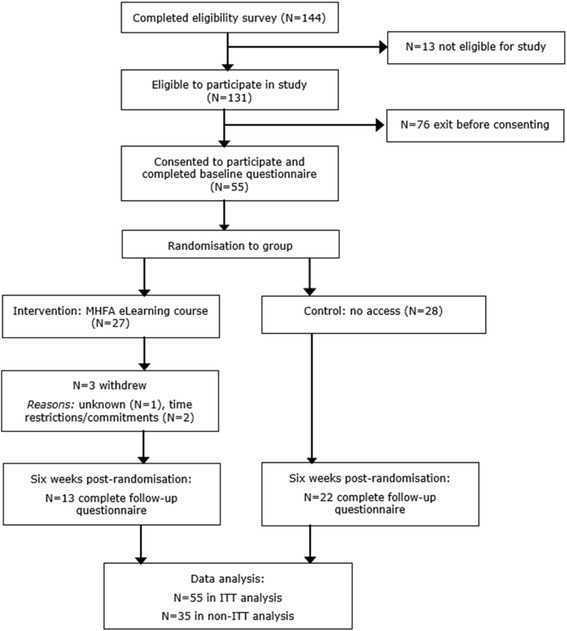
Participant flow through study

Twenty-seven participants were randomised to intervention and 28 to control (Fig. 1). The sample’s mean age was 19.9 ± 3.2 years, with 94.6% (*n* = 52) aged between 18 and 22 years. Two-thirds were female (*n* = 36, 65.5%) and five participants reported undertaking placements or modules relating to psychiatry during their degree. Thirteen (23.6%) reported personal experience of mental health problems, with 40 (72.7%) having had a relative or close friend experience a mental health problem. One participant indicated previous experience of MHFA but did not state when this was. The intervention group reported significantly greater dropout at follow-up (X^2^(1) = 5.49, *p* = .019: see Fig. 1): 14 participants (51.9%) were lost to follow-up, including 3 study withdrawals, compared to only 6 (21.4%) in the control group. There were no baseline differences in demographics or mental health between responders and non-responders (all *p*= > .05).

At baseline, similar proportions of participants in the control group (15/28, 53.6%) and in the intervention group (18/27, 66.7%) correctly identified the most likely mental health condition depicted in the vignette (see Table 3). In both the ITT and non-ITT analyses, there were no pre-post changes in identification of the mental health condition (McNemar’s test *p* = .125 for control, *p* = 1.00 for intervention).

**Table 3:** Descriptive intent-to-treat (ITT: *N* = 55) and non-ITT (*N* = 35) data showing changes in identification of the mental health condition (depression or social anxiety/phobia) described in the vignette^a^

	Vignette			
	Depression		Social anxiety/phobia	
Analysis	Baseline	Follow-up	Baseline	Follow-up
*Intent-To-Treat*				
MHFA eLearning (*n* = 27)	9/10	9/10	9/17	9/17
Control (*n* = 28)	10/16	10/16	5/12	9/12
*Non-ITT*				
MHFA eLearning (*n* = 13)	5/5	5/5	4/8	4/8
Control (*n* = 22)	8/11	8/11	5/11	9/11

^a^Each box shows the number of participants correctly identifying the mental health condition depicted in vignette / total number who viewed the vignette in each trial arm

### Intention to treat analyses

ITT analysis found no main effect for change in mental health first aid intentions between baseline and follow-up (F(1,53) = 3.67, *p* = .06), but there was a significant interaction between time and group (F(1,53) = 16.03, *p* = <.001). Mental health first aid intentions improved in the intervention group (Z = 3.07, *p* = .002), but not in the control group (*p* = 0.09). Confidence to help a friend with mental health problems did increase over time (F(1,51) = 21.22, *p* = <.001) but the group x time interaction failed to reach significance (F(1,51) = 2.98, *p* = .09). There was also a significant main effect for stigma over time (F(1,53) = 217.66, *p* = <.001), and a significant group x time interaction (F(1, 53) = 5.48, *p* = .023). Stigma fell significantly in the intervention group (Z = 2.30, *p* = .021) but not in the control group (See Table 4).

**Table 4:** Intent-to-treat (*N* = 55) analyses for mental health first aid intentions, confidence, personal and perceived stigmatising attitudes

Outcome	Time point	MHFA eLearning (*n* = 27)	Control (*n* = 28)	Effect size (Cohen’s d)
Mental health first aid intentions	Baseline	3.22 (1.36)	3.53 (2.00)	
	Follow-up	4.74 (2.47)	3.00 (1.24)	0.89
Personal stigma	Baseline	5.11 (4.95)	5.57 (4.36)	
	Follow-up	4.14 (4.96)	5.32 (4.57)	0.25
Confidence in helping a friend	Baseline	0.88 (0.57)	0.75 (0.64)	
	Follow-up	1.54 (0.93)	1.00 (0.74)	0.64

### Non-ITT analyses

Non-ITT analyses using only participants who completed baseline and follow-up questionnaires (*N* = 35), showed a broadly similar pattern of results as the ITT analyses but with larger effect sizes (see Table 5). There was a significant overall improvement in mental health first aid intentions over time (F(1,33) = 13.45, *p* = .001) but this was entirely accounted for by the significant interaction between time and group (F(1,33) = 32.39, *p* = <.001). Mental health first aid intentions improved between baseline and follow-up in the intervention group (Z = 3.07, *p* = .002) but there was a non-significant decline in mental health first aid intention scores in controls. There was also a significant main effect (F(1,31) = 47.46, *p* = <.001) for confidence to help a friend and an interaction between time and group (F(1,31) = 14.73, *p* = .001). Confidence scores improved significantly in the intervention group (Z = − 3.01, *p* = .003). In addition stigma reduced over time (F(1,35) = 8.38, *p* = .007) and there was an interaction between time and group (F(1,35) = 4.41, *p* = .043). Stigma significantly declined in the intervention group (Z = − 2.30, *p* = .021) but not in controls (Z = −.748, *p* = .45).

**Table 5:** Non-ITT (*N* = 35) analyses for mental health first aid intentions, confidence, personal and perceived stigmatising attitudes (mean, SD)

Outcome	Time point	MHFA eLearning (*n* = 13)	Control group (*n* = 22)	Effect size (Cohen’s d)
Mental health first aid intentions	Baseline	2.92 (1.32)	3.50 (2.24)	
	Follow-up	6.07 (2.72)	2.81 (1.29)	1.53
Personal stigma	Baseline	5.07 (2.78)	5.18 (4.30)	
	Follow-up	3.07 (2.36)	4.86 (4.53)	0.5
Confidence in helping a friend	Baseline	1.00 (0.57)	0.77 (0.61)	
	Follow-up	2.23 (0.59)	1.10 (0.71)	1.71
Actual mental health first aid actions	Baseline (past six months)	3.00 (2.79)	2.68 (2.80)	
	Follow-up (past six weeks)	7.14 (1.77)	3.36 (2.56)	1.72
Knowledge (*n* = 11)	Baseline	11.45 (1.21)	n/a	
	Follow-up	14.72 (2.14)	n/a	1.88

At follow-up, 7/13 (53.8%) participants in the intervention group reported talking to someone about that person’s mental health problem, compared to 15/22 (68.1%) in the control group, a non-significant difference. For those who reported talking to someone during the six-week trial period, the intervention group reported significantly more mental health first aid actions (Mdn = 7, M = 7.14 ± 1.77) compared to controls (Mdn 2.50, M = 3.36 ± 2.56: Z = − 2.74, *p* = .006).

Intervention group participants who completed the pre-and-post course quiz (*n* = 11) had improved knowledge at follow-up compared to baseline. Participants’ mean knowledge scores at baseline were 11.45 (± 1.21) out of possible maximum score of 20, rising by over three points to 14.72 (± 2.14) at post-course (Z = − 2.94, *p* = .003, *n* = 11).

### Participants’ motivation for participation

Participants gave multiple reasons for participation in the study: the majority of responders (29 out of 35) stated it was because they wanted to gain knowledge about mental health. Other reasons included having personal interests in mental health (*n* = 28); to benefit their studies (*n* = 26); a desire to know how to help someone experiencing a mental health problem (*n* = 26); having personal experience of a mental health problem (*n* = 13); having experienced someone close to them with a mental health problem (*n* = 20); and personal interests in participating in research (*n* = 18).

### Participants’ use and feedback about the MHFA eLearning course

Twenty (74.1%) participants registered and created an account to access the MHFA eLearning course. Fourteen completed the introductory module; 13 completed the depression module; 12 completed the anxiety problems and eating disorders modules; with 11 completing all modules. Participants rated the course as “very easy” (*n* = 7/13), “quite easy” (*n* = 4) or “somewhat easy” (*n* = 1) to navigate, with one finding it “quite difficult” due to its format/presentation. Ten participants reported using the MHFA manual and/or supplementary booklet, and eleven watched and/or listened to all (*n* = 6) or at least one (*n* = 5) of the audio/visual media embedded within the eLearning course. All stated that the course’s content was understandable, suitable and applicable for UK medical students, and did not feel there was any content which could cause harm or offence. Four reported taking more than the estimated 6-to-8 h to complete the course, two were within this estimated time, and three took under six hours.

Positive feedback on the course included its ease of navigation and flexibility for participants to complete it at their own pace, as well as the course being targeted at, and relevancy for, students. Seven participants felt the course was interesting, informative and had learnt new knowledge about mental health, with one stating it “clarified misconceptions”. Eleven participants felt the course had impacted upon their knowledge and understanding of mental health issues; eight participants reported feeling more informed about mental health and how to help someone in need. Almost all (*n* = 12) felt the course had positively impacted on their ability to support someone experiencing a mental health problem and ten participants stated they would recommend the course to fellow medical students.

## Discussion

This study represents the first piloting of the online MHFA course with UK medical students. The results suggest that the MHFA eLearning course improved students’ mental health knowledge, the quality of their intended mental health first aid actions, decreased their stigma about mental health issues, and helped improve their confidence to help someone experiencing a mental health problem. The intervention group was also more likely to report that they had actively used mental health first aid skills and helped a friend with a mental health problem.

To evaluate participants’ recognition of a mental health problem the present study used vignette methodology together with measures and coding schemes used in previous evaluations of MHFA [19]. There was no improvement in ability to identify a common mental health problem but baseline recognition was high and so this could reflect a ceiling effect as found in other studies [4]. Although the ITT analysis for confidence to help a friend failed to demonstrate superiority for the intervention group, non-ITT analysis suggested there was a large and significant benefit for the intervention group. Both ITT and non-ITT analyses demonstrated that the intervention reduced mental health stigma. Mental health stigma is a barrier to help seeking and receiving treatment [38], including in young people [39]. The culture of medical training and practice fosters additional stigmatising beliefs which hinder and prevent medical professionals from seeking out help for a mental health problem [4, 40]. These include fears about being perceived as weak, anticipated impact upon own career development, their peers, colleagues, and patients, and how mental illness reflects their professionalism and fitness-to-practice [7]. Health professionals may project their own stigmatising beliefs and attitudes onto patients, subsequently affecting the quality of care provided and potentially influencing their patients’ treatment, management and future help-seeking behaviour [41]. This also applies to how they respond to a friend experiencing a mental health issue, as social support can influence young people’s help-seeking behaviour [42]. Therefore decreasing stigma associated with mental illness and help-seeking has implications for medical students’ management of their own mental health during their training and future working life. It will also impact on how they support their friends and peers whilst at university, and on how they provide treatment to future patients. The present findings align with previous evaluations of online and CDROM-delivered MHFA [4, 21, 22]. Participants’ improved knowledge, coupled with their improved attitudes towards mental health, can increase trainees’ potential to provide appropriate mental health first aid towards someone in need [4].

The intervention was associated with large and significant improvements in mental health first aid intentions, suggesting that those using the course are better equipped to help others with mental health problems. Crucially participants in the intervention group had provided more actual support to peers following the intervention. However, we did not collect information about *how* they actually provided this support, nor did we gain perspectives from the person who received support from the trained first aider. Previous evaluation of the MHFA eLearning course with financial counsellors [21] did not find any change in intentions, which the authors speculated could have been due to their small sample (*n* = 21); however the present study found significant change with a similar sample size. This may reflect the increased perceived relevance of the course for medical students. The reaffirmation of the ‘ALGEE’ action plan and the use of interactive media-delivered case studies in the course may have allowed participants to better understand the MHFA materials and encouraged skills practice [22]. Comments from participants suggested they valued their improved knowledge about mental health and the ‘ALGEE’ plan taught them a useful way to approach someone they may be concerned about. Although the eLearning course did include interactive tasks to practise and apply new knowledge, some participants mentioned difficulties in rehearsing new skills through eLearning. A ‘blended’ approach involving both online learning and brief face-to-face group training may be beneficial for skills rehearsal [4]: a recent meta-analysis found blended delivery more effective than face-to-face only or eLearning only learning methods [43].

Feedback about the eLearning course was mainly positive: it was said to be interesting and informative, with ten stating they would recommend the course to fellow medical students. As well as improving mental health knowledge, students’ feedback reported that the course helped “clarified misconceptions” about certain areas of mental health, with particular mention to psychosis. Psychotic illnesses are rarer and understandably less understood by the public, and a previous study with Swiss students found they had poorer recognition of ‘true’ symptoms of schizophrenia, in comparison to depression [44]. Despite a large number of students registering their interest, there was a large attrition rate between completing the eligibility questionnaire and consenting to participate. It is speculated that deterring aspects of the study, such as time needed to complete the course on top of medical students’ already heavy workload, might have hindered students’ willingness to complete the course. Some participants took longer than the estimated 6–8 h to complete the course, and also mentioned conflict in completing the course on top of their workload. There may be benefit in timetabling the MHFA course in the medical curriculum as this could improve uptake of the course and thus ensure that more students are equipped to offer effective, informal support for mental health problems.

At baseline, the sample were not overall a group experiencing poor mental health: 70.9% (*N* = 39), 67.2% (*N* = 37) and 74.5% (*N* = 41) of the sample scored within the ‘normal’ threshold range of the depression, anxiety, and stress subscales respectively, and the mean score of each DASS-21 subscale occurred within the ‘normal’ thresholds. There were a minority who were currently experiencing moderate-to-severe symptoms of depression, anxiety and/or stress. Although the MHFA eLearning course is designed to teach trainees how to help someone else in need, trainees may also apply the course’s content to understanding and responding to their own mental health. This is reflected in one participant in the present study, who commented that “*I’ve sought help for a mental health issue. I was aware it might have been a problem but the course helped me recognise the signs*”. Future evaluations of MHFA may also wish to explore the impact of the course upon trainees’ own care and awareness of their mental health.

### Limitations

This short-term study meant we did not explore how participants’ new mental health knowledge and mental health first aid skills impacted on to their actual actions and behaviours in the longer term. However, previous research has found intentions are predictive of actual first aid behaviour [33] and there was a short-term increase in number of reported actions. Using a mixed-methods approach, longer-term follow up could explore how students implement the learnt knowledge and skills into the real world, what helped or hindered them in helping someone with a mental health need, and what factors influence “good” delivery of mental health first aid.

Although the majority of the sample stated that one reason they participated was because they wanted to gain knowledge about mental health, only half of those randomised to the MHFA eLearning group completed the follow-up survey. We have no information about course uptake in the non-completers. Future evaluation of the MHFA eLearning course may wish to be more pragmatic in its design, to be able to fit in with the demands of medical education and to understand the effects of undertaking the course in a real world situation as part of medical training, so that findings may be more generalizable to other British medical schools. A possible option to help support undertaking of the MHFA eLearning course is to include it as part of medical students’ curriculum. In a previous evaluation of the MHFA eLearning course for medical and nursing students, there appeared to be no set deadline for course completion but students were emailed periodic reminders about completing the course [4]. Providing students with a longer period to complete the training and timetabling the course to fit with other academic commitments, may also help to improve uptake.

An already-small sample size was reduced further for the non-ITT analyses, which may have affected the analyses’ statistical power and female participants were over represented. It is important that male students are able to access MHFA training as there is evidence that male students have poorer MHFA skills and may receive less effective help from their male peers for their own mental health difficulties [10]. Even with reminder emails, a third (*n* = 17) did not complete the follow-up questionnaire; this clearly further reduced our already-small sample. Notably, there were more participants in the intervention group who did not complete the follow-up questionnaire. Usage data shows that the majority (11 out of 13) of the intervention group participants who completed the follow-up survey were those who completed the whole course. Those participants who did not complete the course may have been reluctant to complete the follow-up questionnaires. There was an unequal gender balance in the sample, which was not representative of the medical student cohort at UoN (56% female). The majority of these reported having used the MHFA manual and supplementary reading material, and engaged with the interactive tasks within the course. However we do not know the opinions of those who did not complete the course; it is possible that there were certain aspects of the course which influenced their decision to engage with it (e.g. content, time needed to complete it).

The previous evaluation with medical students was an uncontrolled trial, where students in the two groups both received MHFA, either through group face-to-face delivery or the eLearning format [4]. The present pilot study did not use an active control, such as a freely available mental health resource, which was a limitation of the study. After the six week study period, control group participants were debriefed via email. We were not resourced to offer the control group access to the MHFA eLearning course but they did receive detailed information about in-person and online mental health services and resources, including the freely-available MHFA action plan guidelines that are taught within the MHFA eLearning course.

## Conclusion

The results provide preliminary support for the effectiveness and feasibility of the MHFA eLearning course for UK medical students. The eLearning course increased the quality of medical students’ mental health first aid intentions, improved their confidence in their ability to help a friend with mental health problems and reduced mental health stigma. Feedback about the MHFA eLearning course was encouraging, and identified some potential areas for improvement. Future research should explore the impact of embedding the MHFA course within the medical curriculum and identify barriers to uptake in a larger randomised trial.

## Abbreviations

ALGEE: The five-step Mental Health First Aid action plan (approach the person, assess and assist with any crisis; listen non-judgementally; give support and information; encourage the person to seek professional help; encourage other support strategies)
DASS-21: Depression, Anxiety and Stress scales, 21-item version
DSS: Depression Stigma Scale
ITT: Intention-to-treat analysis
MHFA: Mental Health First Aid course
UoN: the University of Nottingham
